# A Comprehensive Evaluation of Colonic Mucosal Isolates of *Sutterella wadsworthensis* from Inflammatory Bowel Disease

**DOI:** 10.1371/journal.pone.0027076

**Published:** 2011-10-31

**Authors:** Indrani Mukhopadhya, Richard Hansen, Charlotte E. Nicholl, Yazeid A. Alhaidan, John M. Thomson, Susan H. Berry, Craig Pattinson, David A. Stead, Richard K. Russell, Emad M. El-Omar, Georgina L. Hold

**Affiliations:** 1 Gastrointestinal Research Group, Division of Applied Medicine, University of Aberdeen, Foresterhill, Aberdeen, United Kingdom; 2 Child Health, University of Aberdeen, Royal Aberdeen Children's Hospital, Foresterhill, Aberdeen, United Kingdom; 3 Aberdeen Proteomics Group, Institute of Medical Sciences, University of Aberdeen, Foresterhill, Aberdeen, United Kingdom; 4 Department of Paediatric Gastroenterology, Royal Hospital for Sick Children, Glasgow, United Kingdom; Charité-University Medicine Berlin, Germany

## Abstract

Inflammatory bowel disease (IBD) arises in genetically susceptible individuals as a result of an unidentified environmental trigger, possibly a hitherto unknown bacterial pathogen. Twenty-six clinical isolates of *Sutterella wadsworthensis* were obtained from 134 adults and 61 pediatric patients undergoing colonoscopy, of whom 69 and 29 respectively had IBD. *S. wadsworthensis* was initially more frequently isolated from IBD subjects, hence this comprehensive study was undertaken to elucidate its role in IBD. Utilizing these samples, a newly designed PCR was developed, to study the prevalence of this bacterium in adult patients with ulcerative colitis (UC). *Sutterella wadsworthensis* was detected in 83.8% of adult patients with UC as opposed to 86.1% of control subjects (p = 0.64). Selected strains from IBD cases and controls were studied to elicit morphological, proteomic, genotypic and pathogenic differences. This study reports Scanning Electron Microscopy (SEM) appearances and characteristic MALDI-TOF MS protein profiles of *S. wadsworthensis* for the very first time. SEM showed that the bacterium is pleomorphic, existing in predominantly two morphological forms, long rods and coccobacilli. No differences were noted in the MALDI-TOF mass spectrometry proteomic analysis. There was no distinct clustering of strains identified from cases and controls on sequence analysis. Cytokine response after monocyte challenge with strains from patients with IBD and controls did not yield any significant differences. Our studies indicate that *S. wadsworthensis* is unlikely to play a role in the pathogenesis of IBD. Strains from cases of IBD could not be distinguished from those identified from controls.

## Introduction

Inflammatory bowel disease (IBD) is an idiopathic inflammatory disorder that is comprised of two major phenotypes, Crohn's disease (CD) and ulcerative colitis (UC). The understanding of its aetiopathogenesis has taken rapid strides in the last decade, with current investigations focusing heavily on aberrations in host-microbe interactions at the luminal intestinal surface. Genetic defects in pathogen recognition and primary handling of microbes by the innate immune system compounded with distinct changes in the luminal microbiome or dysbiosis form the current backbone of this pathogenic hypothesis [1], [2]. Despite this, researchers in the field have been striving to identify and delineate a solitary micro-organism that can explain the initiation and perpetuation of this chronic inflammatory process.

In this regard, anaerobic and microaerophilic bacteria residing in the intestinal lumen have often been the neglected species, primarily on account of the intrinsic difficulty in culturing and isolating these organisms by using traditional microbiological techniques. Molecular studies have demonstrated that a substantial proportion of bacterial species (up to 30–40% of dominant species) in patients with active IBD belong to phylogenetic groups that are unusual when compared to healthy subjects [3], [4]. With this premise in mind, our laboratory has focused on enhanced and improved bacteriological conditions for the optimum growth of microaerophilic bacteria from colonic biopsy samples [5], [6]. In our pilot studies we noted the unusual preponderance of the rare microaerophilic Gram negative bacterium *Sutterella wadsworthensis* from cultures of biopsy samples from patients with IBD. This unusual organism has been encountered before by Mangin *et al*. who used 16S rRNA gene sequencing to create molecular inventories of the dominant fecal bacteria in four CD patients and four controls. They found that bacterial species which were not commonly dominant in healthy individuals were over-represented in CD. One of these species included *S. wadsworthensis*, which belonged to the dominant microbiota of one of the four CD patients [7].

These bacteria were first reported when performing biochemical characterization and susceptibility testing of *Campylobacter gracilis* clinical isolates from patients with diverse infections of the GI tract [8]. These organisms could be differentiated from *C. gracilis* mainly by their bile resistance and cell wall fatty acid patterns. 16S rRNA gene sequencing confirmed that these unusual organisms were not related phylogenetically to any of the *Campylobacters*, including *C. gracilis*, with the closest taxa belonging to unrelated aerobes. This was ratified by a subsequent report which demonstrated that the majority of *S. wadsworthensis* strains were isolated from GI infections, only occasionally being isolated from non-abdominal specimens, and were more likely to be involved in serious infections than *C. gracilis* [9]. The supposition was that *S. wadsworthensis* was a putative human pathogen. Three other species, *Sutterella stercoricanis*, *Sutterella morbirenis and Sutterella parvirubra* belonging to the same genera have subsequently been identified from canine and human feces [10], [11].

The role of this group of bacteria has not been clearly elucidated in the aetiopathogenesis of IBD. This study has for the first time outlined the role of *S. wadsworthensis* in patients with IBD and performed a comprehensive phenotypic, genotypic, proteomic and pathogenetic characterization of this bacterial species, which will serve as a useful benchmark for future studies.

## Methods

### Study subjects, specimen collection and processing

Adult patients were recruited from the Department of Gastroenterology at the Aberdeen Royal Infirmary. These subjects were recruited for a previous study looking at the role of enterohepatic *Helicobacter* in UC [5]. A total of sixty-nine patients with a diagnosis of UC made on the basis of histology of colonoscopic biopsies were recruited and assessed. Sixty-five healthy controls were contacted prior to their index colonoscopy as part of the bowel cancer screening programme and recruited for the study if they had documented absence of both macroscopic and microscopic inflammation. Children were recruited from the Departments of Paediatric Gastroenterology, Hepatology and Nutrition at the Royal Aberdeen Children's Hospital and the Royal Hospital for Sick Children (Yorkhill), Glasgow as part of an ongoing study to investigate the role of microaerophilic colonic microbiota in *de-novo* paediatric IBD (Bacteria in Inflammatory bowel disease in Scottish Children Undergoing Investigation before Treatment: BISCUIT study). Twenty-nine paediatric patients with newly-presenting, treatment naïve IBD and thirty-two paediatric controls undergoing routine colonoscopy were included in this present study [12]. The extent and severity of disease was assigned using the Montreal criteria [13]. Subjects were excluded if they received antibiotics within three months prior to recruitment. Mucosal colonic biopsies were obtained during the colonoscopy procedures. One to two biopsies were used for culture work and the rest were then transferred to a −80°C freezer for storage pending DNA extraction and analysis.

### Ethics

Ethical approval for the study was granted by the North of Scotland Research Ethics Service, UK (reference numbers 04/S0802/8 and 09/S0802/24). Written informed consent was obtained from all adult subjects and from the parents of all paediatric subjects in the study. Informed assent was also obtained from older children who were deemed capable of understanding the nature of the study.

### Bacterial strains

#### Sutterella wadsworthensis strains

A total of twenty-seven *S. wadsworthensis* strains were used in this study. The type strain, DSM 14016 ( = ATCC 51579) was obtained from the German Collection of Microorganisms and Cell Cultures (DSMZ). Another twenty-six *S. wadsworthensis* strains obtained during the course of this study were also examined.

#### Other bacterial strains

Twenty-eight other bacterial strains were used in this study, obtained from international culture collections as well as from clinical and environmental sources: *Campylobacter jejuni* (NCTC 11351), *Campylobacter upsaliensis* (NCTC 11540), *Campylobacter fetus* (NCTC 10842), *Campylobacter lari* (NCTC 11352), *Campylobacter coli* (clinical isolate), *Campylobacter concisus* (clinical isolate), *Helicobacter pylori* (ATCC 700392), *Helicobacter hepaticus* (ATCC 51449), *Helicobacter cholecystitis* (ATCC 700242), *Helicobacter canis* (ATCC 51402), *Helicobacter canadensis* (ATCC 700968), *Escherichia coli* (NCIMB 12201), Pseudomonas *fluorescences* (clinical isolate), *Pseudomonas aeruginosa* (ATCC 27853), *Shigella sonnei* (25931 clinical isolate), *Proteus mirabilis* (NCTC 3177), *Proteus vulgaris* (NCTC 4157), *Salmonella enteritidis* (NCTC 12694), *Salmonella typhimurium* (NCIMB 13284), *Staphylococus aureus* (NCIMB 12702), *Bacillus cereus* (ATCC 10876), *Enterococcus faecalis* (NCIMB 13280), *Enterobacter aerogenes* (NCIMB 10102), *Listeria monocytogenes* (clinical isolate), *Fusobacterium nucleotum* (clinical isolate), *Klebsiella pneumonia* (NCIMB 13281), *Acinetobacter calcoaceticus* (clinical isolate), *Yersinia fredericksenii* (NCIMB 2124), *and Aeromonas caviae* (clinical isolate).

### *Sutterella wadsworthensis* growth conditions

*S. wadsworthensis* isolates were obtained by culturing 1–2 mucosal biopsy samples on five selective plates, the details of which are listed in Table 1. Biopsies were first ground in brucella broth before the resultant suspension was added to the plates. 50 µl was added to each plate with the exception of the filtered blood plate where 200 µl was first passed through a 0.45 µm filter. Cultures were incubated in a micro-aerophilic atmosphere, comprising of 5.9% oxygen, 7.2% carbon dioxide, 3.6% hydrogen and 83.3% nitrogen at 37°C. This atmosphere was generated using Anoxomat® Atmosphere Generating System, from Mart® Microbiology b.v. (9200 JB Drachten, Netherlands). Plates were reviewed twice weekly for up to one month. Any bacterial isolate deemed Gram-negative and oxygen sensitive (by virtue of failed subculture in room air) was identified by sequencing of the 16S rRNA gene and sequence search on NCBI BLAST (http://blast.ncbi.nlm.nih.gov/Blast.cgi).

**Table 1:** Culture media used in the study.

Media Name	Base	Additives	Volume of biopsy suspension added	Reference
Blood agar	Blood Agar	Nil	50μmol	-
Blood Agar	Blood Agar	Nil	200μmol through 0.45μm filter	-
Skirrow	Blood Agar	Polymyxin B, Trimethoprim, Vancomycin	50μmol	[27]
CVA	Blood Agar	Amphotericin, Cefoperazone, Vancomycin	50μmol	[28]
Helicobacter	Blood Agar	Amphotericin, Bacitracin, Nalidixic acid, Polymyxin B, Vancomycin	50μmol	[29]
Campylobacte	Blood Agar	Trimethoprim, Vancomycin	50μmol	[30]

### Genotypic Characterization

#### DNA Extraction

Genomic DNA was extracted from the colonic mucosal biopsies using the QIAamp DNA Mini Kit (Qiagen, Crawley, UK) according to an established modification of the manufacturer's instructions, optimised in-house for colonic biopsy tissue as described previously [5].

#### *S. wadsworthensis* specific primer design

For the design of a new *S. wadsworthensis* -specific primer, 16S rRNA gene sequences of all the *S. wadsworthensis* strains and other related bacterial strains were obtained from the NCBI database (http://www.ncbi.nlm.nih.gov) for multiple alignment. Nearly full length 16S rRNA sequences of *S. wadsworthensis* strains isolated from clinical specimens in this study were also used for the alignment analysis. Several primer sequences were initially designed by using the Primer 3 software [14] and then the most suitable one was identified manually using the BioEdit software package (version 7.0.5.3) (http://www.mbio.ncsu.edu/BioEdit/bioedit.html). The downloaded *S. wadsworthensis* specific sequences were aligned with the newly designed primer sequences and the ones that matched to the highly conserved 16S rRNA gene sequences of the target species but were variable among the other bacterial species, were selected. The 20 base pair *S. wadsworthensis* specific primer sequences designed (SWF and SWR) are outlined in Table 2. The primer sequences were then subjected to BLAST analysis against all the other sequences within the BLAST database to ensure that at least one of the primers did not share any identical sequence with any microorganisms other than S. *wadsworthensis*.

**Table 2:** Sequences of PCR primers designed in this study.

Primer name	Sequence	Reference
SWF	5′- GAC GAA AAG GGA TGC GAT AA - 3′	This study
SWR	5′- CTG GCA TGT CAA GGC TAG GT- 3′	This study

#### Optimization of *S. wadsworthensis* PCR assay

The specificity of the newly developed *S. wadsworthensis* primer pair was tested against a wide panel of bacterial species as listed earlier in the methods section. The PCR assay was tested using 40 ng of bacterial DNA in a 50 µl reaction mixture consisting of 10 pmol of each primer (SWF and SWR [Sigma-Aldrich, UK]), 1X PCR buffer (Roche, UK), 250 nM of each deoxy-nucleotide-triphosphate (Bioline, UK), 2 mM MgCl_2_ (Roche, UK) and 1 U of Taq polymerase (Roche,UK). The sensitivity of the PCR assay was determined by serially diluting a known quantity of target DNA (50 ng/µl to 0.005 pg/ µl) to detect the minimum concentration that would yield a visible amplicon after gel electrophoresis.

Restriction fragment length polymorphism (RFLP) analysis was also done using the 555 bp *S. wadsworthensis*-specific PCR products using *EcoR* I and *Hha* I enymes to confirm that the PCR products were from a single bacterial species.

#### Sequence Analysis

Sequencing was done on an Applied Biosystems model 3730 automated capillary DNA sequencer using the *S. wadsworthensis* - specific primers SWF and SWR or 16S rRNA specific universal bacterial primers for the whole gene amplification of the *S. wadsworthensis* strains. The sequences obtained were compared to those of the National Center for Biotechnology Information GenBank database using the basic local alignment search tool (BLAST) search program. Multiple alignments and phylogenetic analysis were performed using Bioedit (http://www.mbio.ncsu.edu/BioEdit/bioedit.html) (version 7.0.5.3) and a dendogram was constructed using MEGA version 4 software [15]. The evolutionary distances were calculated according to Kimura's two-parameter model. A phylogenetic tree was inferred using the neighbor-joining algorithm, and the tree topology was statistically evaluated by 1,000 bootstrap resamplings.

#### GenBank Sequence Submission

All 16S rRNA gene sequences derived from the sequencing of *S. wadsworthensis-* specific PCR products were submitted to GenBank with the accession numbers from JN664092-JN664117.

### Phenotypic Characterization

Basic biochemical tests including Gram staining, catalase, urease and oxidase tests were conducted using conventional manual methods to characterize the isolates. The strains were further characterized using the API Campy commercial biochemical kit according to the manufacturer's instructions (BioMerieux, La Balme Les Grottes, France). The purpose of using the API kits in this study was not to identify or name the bacteria, but to use the biochemical tests to further characterize the *S. wadsworthensis* strains. By comparing the resultant biochemical profiles, any potential differences between the strains might be elicited. Of all the API kits available it was assumed that API Campy would yield the best results, as *S. wadsworthensis* is phenotypically similar to *Campylobacter* species.

### Scanning Electron Microscopy (SEM)

*S. wadsworthensis* strains were harvested for SEM. Bacterial cells were suspended in 2% Glutaraldehyde in 0.1 M sodium Cacodylate buffer (pH 7.2) for 3 hours to be fixed. The fixed cell suspensions were then applied to glass coverslips coated with poly-L-lysine and allowed to adhere for five minutes. Coverslips were then rinsed with water and post-fixed with 1% Osmium tetroxide (OsO4) for 30 minutes. They were next rinsed with water and dehydrated through a graded ethanol series (60%, 80%, and 90%) for 10 minutes each. The sample was then left for 30 minutes in absolute ethanol with ethanol changes every 10 minutes. The coverslips were kept in Hexamethyldisilazane (HMDS) Sigma® for 10 minutes, and left to dry overnight. Next day, the specimen was coated with gold using automated sputter coater EMITECH K550 (Emitech limited, Ashford, Kent) and samples were transferred to view *S. wadsworthensis* using SEM.

### Proteomic Characterization

To complement the phenotypic and genotypic methods, matrix-associated laser desorption/ionisation - time of flight mass spectrometry (MALDI-TOF MS) was performed to further characterize selected *S. wadsworthensis* strains.

#### Sample preparation

A total of 11 bacterial strains were analyzed by MALDI-TOF MS: eight *S. wadsworthensis* isolates (4 strains obtained from healthy controls and 4 strains from IBD patients), the *S. wadsworthensis* type strain DSM 14016, *C. jejuni* and *C. concisus* strains. Approximately 5-10 mg (half a plastic loop) of bacterial cells were harvested from fresh blood agar plates and proteins were extracted as described by Alispahic *et al.* (2010) [16]. The protein extract (supernatant) was diluted, with 30% acetonitrile in 0.1% trifluoracetic acid (Applied Biosystems, UK) in water. Subsequently, 1 µl of diluted supernatant was mixed with 1 µl of matrix solution (10 mg HCCA (α-cyano-4-hydroxycinnamic acid) matrix (Bruker Daltonics, Germany) to 1 ml of a solution comprising: 500 µl acetonitrile, 475 µl UHQ water and 25 µl trifluoroacetic acid). 1 µl of the sample-matrix mixture was pipetted onto a MALDI ground steel target plate (Bruker Daltonics) and left to dry on air.

#### MALDI-TOF MS

Mass spectra were acquired using an ultrafleXtreme™ series MALDI-TOF MS (Bruker Daltonics), equipped with a 1 kHz smartbeam-II™ nitrogen laser (λ = 337 nm). Spectra were recorded using the linear positive mode for masses in the range of 2 kDa to 20 kDa. The parameter settings were as follows: ion source 1, 25 kV; ion source 2, 23.85 kV; lens, 6.5 kV; pulse ion extraction time, 160 ns. A low-mass gate of 800 Da was used. Insulin, ubiquitin, cytochrome C and myoglobin were used as external calibrants, enabling a mass accuracy of ±0.5 parts per 1000. Laser power and the number of laser shots per sample were varied by the operator for optimal performance.

#### Data analysis

Mass spectra were processed prior to visual inspection using FlexAnalysis 3.3 software (Bruker Daltonics). This included smoothing, baseline subtraction and intensity normalisation. MALDI BioTyper software 2.0 (Bruker Daltonics) was used to generate pseudo gel view representations of each spectrum. The mass spectra and corresponding pseudo gel images for each bacterial strain were then visually inspected and compared.

### Preparation of monocytes and *S. wadsworthensis* (whole cell) stimulation studies

Whole venous blood was collected from six adult healthy individuals who were not on any medications. The blood was diluted 1:1 in RPMI 1640 (Sigma-Aldrich), layered onto Histopaque-1077 (Sigma-Aldrich), and centrifuged at 800×g for 30 min. Peripheral blood mononuclear cells were removed from the interface and monocytes were prepared as published earlier [17]. The effect of *S. wadsworthensis* whole cell preparations was assessed by stimulating the monocytes for up to 24 hours. Seven *S. wadsworthensis* strains (4 strains obtained from healthy controls and 3 strains from IBD patients) were used in this study along with an *E. coli* (S1041) as a positive control. Unstimulated monocytes were used as a negative control.

### Cytometric bead array

In order to assess whether the whole cell preparations induced an inflammatory response, levels of TNF-α in culture supernatant were measured at the different time points using a human inflammatory cytokine cytometric bead array (CBA;BD Biosciences). Samples were analyzed on a multifluorescence BD FACSCalibur™ flow cytometer using BD CellQuest™software and BD™ cytometric bead array software. Standard curves were generated using FCAP Array Software™. Sample results were then calculated by comparing sample CBA results to the respective standard curve. All CBA work was performed in duplicate and cytokine levels induced by stimulation were calculated by subtracting unstimulated cytokine levels from each stimulated value and expressing TNF-α levels as a fold-change relative to TNF-α levels derived from *E. coli* stimulations.

### Statistical Analysis

Statistical analysis was performed using the Pearson Chi Squared, 2-tailed test or the Fisher's exact test wherever appropriate, utilizing Graph Pad software (San Diego, CA).

## Results

### Assessment of *S. wadsworthensis* prevalence in mucosal colonic biopsies

#### Clinical information of the study population

The mean age of the sixty-nine patients with UC was 45.6±27.8 years and 46.4% were male as opposed to the sixty-five control patients whose mean age was 64.2±5.6 years and 59.3% of these subjects were male. There was a statistically significant difference in age between the UC cohort and the control group (p<0.0001). At the time of colonoscopy the adult patients were classified as extensive (24.6%), left sided (63.8%) and proctitis (11.6%) according to the Montreal criteria. During recruitment 10.1% of patients were in clinical remission (S0), 23.2% had mild UC (S1), 46.4% had moderate UC (S2) and the remaining 20.3% had severe UC (S3). A total of 115 biopsy sites were analyzed from UC patients. Twenty-four (34.8%) subjects had a single site analyzed and forty-five (65.2%) had more than one biopsy site assessed. A single biopsy site was analyzed from each control subject. The adult patients served as clinical sources for the isolation of *S. wadsworthensis.* The newly developed PCR was utilized to detect the presence of *S. wadsworthensis* from mucosal biopsies from these patients.

The paediatric IBD patients (n = 29, male 62.1%) had a median age of 12.2 years (interquartile range, IQR, 9.8, 14.1 years) at the time of recruitment out of which fifteen had CD, nine UC and five IBD-unclassified. The paediatric control patients (n = 32, male 78.1%) had a mean age of 10.8 years (IQR, 8.3, 12.7 years) at the time of index colonoscopy. The paediatric cohort was primarily utilized as a source for isolation of *S. wadsworthensis*, which was then utilized to develop and standardize the detection PCR.

A total of twenty-six *S. wadsworthensis* strains were isolated from the mucosal colonic biopsy samples of the above mentioned study populations. The majority of the clinical strains were obtained from paediatric patients, ten from control and seven from IBD patients. Six strains were obtained from adult IBD patients and three from adult control patients.

#### Evaluation of the sensitivity and specificity of the newly developed *S. wadsworthensis* -specific PCR assay

**Figure 1:**
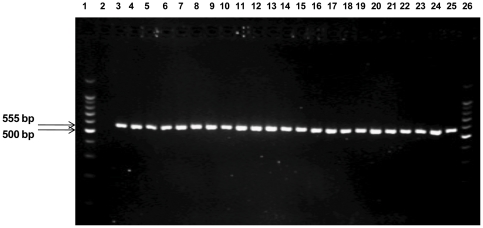
Amplification of *S. wadsworthensis* DNA from reference and clinical strains using the SW-F and SW-R primers. The assay amplified a product of ≈555 bp in size. Lane 1:100 bp marker, Lane 2: negative control (no DNA), Lane 3: *S. wadsworthensis* DSM 14016, Lane 4 – Lane 25: *S. wadsworthensis* isolate SW1, SW2, SW4, SW5, SW6, SW7, SW8, SW9, SW10, SW11, SW12, SW13, SW14, SW15, SW16, SW17, SW18, SW19, SW20, SW21, SW22, SW23, Lane 26:100 bp marker.

**Figure 2:**
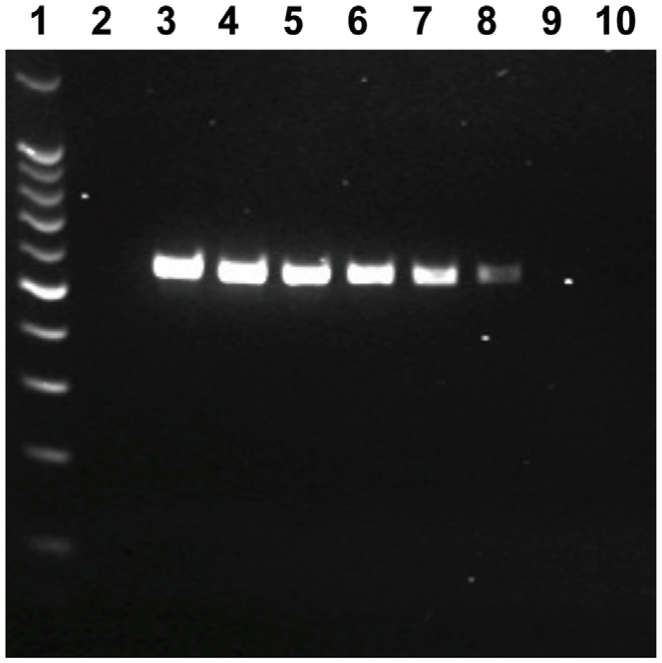
Limit of detection for the newly developed *S. wadsworthensis*-specific nested PCR assay. The PCR assay was able to detect 0.5 pg of *S. wadsworthensis* DNA. Lane 1: 100 bp marker, Lane 2: negative control (no DNA), Lane 3: *S. wadsworthensis* DNA 50 ng/µl, Lane 4: *S. wadsworthensis* DNA 5 ng/µl, Lane 5: *S. wadsworthensis* DNA 0.5 ng/µl, Lane 6: *S. wadsworthensis* DNA 50 pg/µl, Lane 7:*S. wadsworthensis* DNA 5 pg/µl, Lane 8: *S. wadsworthensis* DNA 0.5 pg/µl, Lane 9: *S. wadsworthensis* DNA 0.05 pg/µl, Lane 10: *S. wadsworthensis* DNA 0.005 pg/µl.

Twenty-seven strains of *S. wadsworthensis* and twenty-eight other bacterial strains representing a broad spectrum of bacterial species were screened to test for the specificity of the newly designed species-specific primers. The primer pair produced an intense band of ≈555 bp from all the *S. wadsworthensis* strains (Figure 1). No amplicon was observed from any of the other bacterial strains tested. For detection of *S. wadsworthensis* DNA from mucosal biopsy samples a nested PCR approach was taken where the first round PCR was done using universal bacterial primers (27F and 1492R) [18] followed by a second round of PCR using the *S. wadsworthensis*–specific SWF and SWR primers. The optimal thermal cycling conditions required to obtain specificity for the *S. wadsworthensis*-specific PCR were: 94°C for 5 minutes, 30 cycles of 94°C for 30 seconds, 58°C for 30 seconds, and 72°C for 2 minutes, followed by 72°C for 15 minutes. The optimized PCR was able to detect 0.5 pg/µl of *S. wadsworthensis* DNA (Figure 2).

The RFLP analysis of all the *S. wadsworthensis*–specific PCR products also showed the expected banding pattern of 327 bp and 228 bp for *EcoR* 1 digestion and 420 bp and 135 bp for *Hha* I digestion respectively.

#### Prevalence of *S. wadsworthensis* in adults with UC using species-specific PCR

All the adult mucosal biopsy samples were screened using the newly developed and optimized *S. wadsworthensis*-specific PCR. *S. wadsworthensis* was detected in fifty-seven of the sixty-nine subjects with ulcerative colitis and fifty-six of the sixty-five controls. The prevalence of *S. wadsworthensis* in the UC population was 83.8% which was similar to that in the controls, 86.1% (p = 0.64). There was no statistically-significant difference in the prevalence of *S. wadsworthensis* in male subjects as opposed to female patients. Furthermore, no age related differences could be demonstrated. Looking specifically at patients with UC, no statistically-significant differences were noted in the prevalence of *S. wadsworthensis* with respect to extent or severity of UC according to the Montreal classification.

#### Phylogenetic Analysis

**Figure 3:**
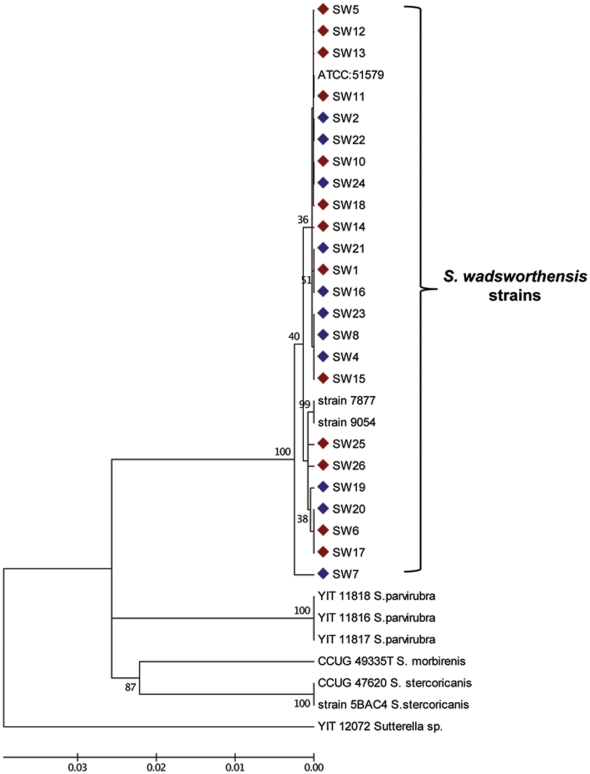
Phylogenetic tree constructed using nearly full-length (∼1400 bp) sequences of the 16S rRNA gene of *S. wadsworthensis* strains from UC and controls alongside other *Sutterella* sequences available in GenBank. The evolutionary history was inferred using the Neighbor-Joining method. The percentage of replicate trees in which the associated taxa clustered together in the bootstrap test (1000 replicates) are shown next to the branches. The tree is drawn to scale, with branch lengths in the same units as those of the evolutionary distances used to infer the phylogenetic tree. The *S. wadsworthenis* isolates from IBD cases are marked in red and those from controls are marked in blue.

A phylogenetic tree was constructed based on nearly full-length (∼1400 bp) 16S rRNA gene sequences of the *S. wadsworthensis* strains isolated from this study and sequences of *S. wadsworthensis* strains and other *Sutterella* species available in GenBank (Figure 3). All the *S. wadsworthensis* sequences derived from this study clustered closely with the other *S. wadsworthensis* sequences from GenBank and presented the highest (99–100%) sequence identity to the *S. wadsworthensis* type strain ATCC 51579. The *S. wadsworthensis* strains from different geographical locations (strain ATCC 51579, strain WAL 7877 and strain WAL 9054 originating from USA and the strains from this study being from the UK) did not cluster into different lineages. Also, *S. wadsworthensis* sequences analyzed from the UC and the healthy controls did not cluster into separate groups in the dendrogram. The GenBank derived sequences from the *S. parvirubra*, *S. stercoricanis and S. morbirenis* strains each grouped into different clusters in the dendrogram confirming different species.

### Characterization of *S. wadsworthensis* isolates to detect strain difference

As the PCR results revealed almost universal prevalence of *S. wadsworthensis* in both IBD cases and controls, we hypothesised that there may be differences between the strains isolated from the two groups and so went on to study their phenotypic, morphological, proteomic and pathogenic profiles.

#### Phenotypic Characterization

*S. wadsworthensis* was found to be a Gram-negative pleomorphic rod shaped organism with different morphological structures seen on Gram stain, even though the *S. wadsworthensis* cultures were pure. All *S. wadsworthensis* strains tested negative for catalase, urease and oxidase tests. The API Campy kit results showed that all the *S. wadsworthensis* strains tested (9 clinical isolates and the type strain) were positive for the EST, ArgA and AspA tests, indicating they possess the enzymes esterase, L-arginine arylamidase and L-aspartate arylamidase respectively. The only discrepant result between the *S. wadsworthensis* strains was for the HIP test; four strains, including the type strain, tested positive and six strains were found to be negative. This tested the ability of the bacteria to hydrolyse sodium hippurate. A positive result indicated the presence of the enzyme hippurate hydrolase. All the assimilation tests on the second half of the strips were negative for all *S. wadsworthensis* strains.

#### SEM

**Figure 4:**
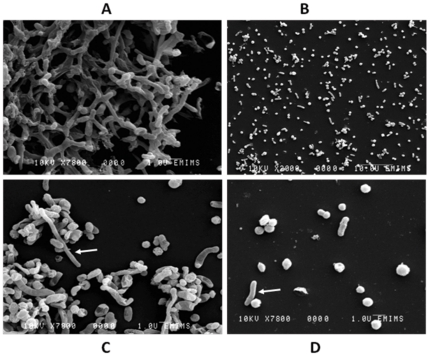
Scanning electron microscopy (SEM) images of *S. wadsworthensis* showing different morphologies for this organism. Small and large, filamentous and helical bacteria forms were observed. A) *S. wadsworthensis* cells formed as a lattice B) Different *Sutterella* cell morphologies C) Filamentous bacterial form D) Helical bacterial form.

Under conventional light microscopy, there were clearly two different morphologies for *S. wadsworthensis*. For this reason, an intensive literature search for *S. wadsworthensis* electron micrographs was done. However, there were no published electron micrograph images available to answer the morphology question. Due to this, two *S. wadsworthensis* strains (SW9 from control and SW13 from IBD) were selected for SEM. The SEM images clearly indicated lattice formation (Figure 4A) and two distinct morphologies of this bacterium: a long rod (nearly 4μm) and a short cocco-bacillus (nearly 1μm) (Figure 4B). Interestingly, there were also cells with filamentous shape (Figure 4C) and a helical form was also observed (Figure 4D).

#### MALDI-TOF MS

Matrix-associated laser desorption/ionization – time of flight mass spectrometry (MALDI-TOF MS) is a well-established technique in proteomics for the analysis of proteins and peptides. Profiling of proteins by MALDI-TOF MS has been developed as a rapid and sensitive technique for microorganism identification [16]. In the MALDI Biotyper system developed by Bruker Daltonics, MALDI-TOF mass spectra of small proteins (range 2–20 kDa) acquired directly from smears of cells or their extracts are compared with reference spectra in the MALDI Biotyper database. The most recent MALDI Biotyper 2.0 database available (version 3.1.2, dated 28 January 2011, containing reference spectra for 3740 microorganisms) did not contain any information on *S. wadsworthensis*. The purpose of MALDI-TOF MS in this study, as with the API testing kit, was not to identify the bacterial strains but to further characterize them and elicit any differences between them.

**Figure 5:**
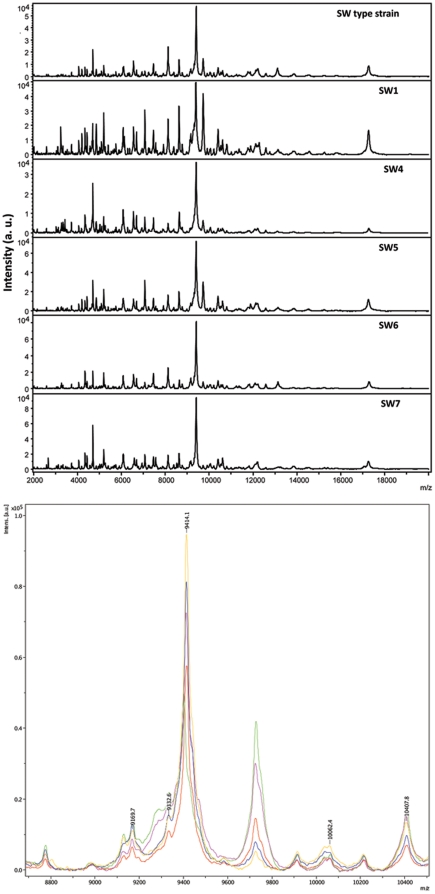
Mass spectra obtained for *S. wadsworthensis* strains. (A) Spectra obtained for 6 *S. wadsworthensis* strains: *S. wadsworthensis* type strain, SW1, SW4, SW5, SW6 and SW7. All of the *S. wadsworthensis* strains conform to a common pattern. a.u. arbitary unit; m/z, mass-to-charge ratio; Da, Daltons; (B) Overlaid spectra of five *S. wadsworthensis* strains overlaid. This is a close-up of the mass spectra from 8,700 Da to 10,500 Da. It shows how closely each *S. wadsworthensis* mass spectrum, aside from intensity, matches the others. a.u., arbitary unit; m/z, mass-to-charge ratio; Da, daltons.

Out of the eleven strains tested, triplicate samples of the first six bacterial strains analyzed by MALDI-TOF MS were analyzed to assess how reproducible the method was. All three replicates for these six strains produced almost identical results, so it was decided that obtaining a single mass spectrum for each of the remaining five strains would be sufficient. The remaining five strains were analyzed and the mass spectra for all 11 strains were obtained. It was immediately apparent that there was a characteristic *S. wadsworthensis* pattern that all of the strains conformed to (Figure 5A). Notably there was a dominant peak at approximately 9400Da which was common to all *S. wadsworthensis* strains (Figure 5B). The *Campylobacter* strains on the other hand, produced different patterns of peaks, with *C. jejuni* producing a dominant double peak at approximately 9500Da and 10300Da (data not shown).

#### Monocyte challenge

A human monocyte challenge system was used to assess the immunogenicity of different *S. wadsworthensis* strains. A survival test was performed to examine the ability of *S. wadsworthensis* strains to survive when exposed to the oxygen present in the monocyte stimulation experimental setup. This showed that >50% of *S. wadsworthensis* cells survived for four hours. Seven *S. wadsworthensis* strains and an *E. coli strain* were inoculated onto monocytes that were obtained from six healthy volunteers. Levels of TNF-α were measured at 4 hours of stimulation. All *S. wadsworthensis* strains induced TNF-α cytokine production from the human monocytes. Compared to the *E. coli* strain used *S. wadsworthensis* strains induced higher levels of TNF-α production over the first 4 hours although subsequent measurements at 24 hours indicated that TNF-α levels remained detectable in *E. coli* stimulated monocytes but that TNF-α was not detectable in *S. wadsworthensis* stimulated wells, most likely because of the death of *S. wadsworthensis* organisms (Table 3).

**Table 3:** TNF-α levels (depicted as percentage) derived from human monocytes (N = 6) exposed to whole cell preparations of various *S. wadsworthensis* strains.

	SW1	SW2	SW3	SW7	SW8	SW9	SW13
**Volunteer A**	3	407	62	43	64	92	128
**Volunteer B**	0	651	184	108	200	178	NT
**Volunteer C**	17	120	908	125	120	120	NT
**Volunteer D**	747	924	197	360	350	541	NT
**Volunteer E**	163	625	117	237	237	429	NT
**Volunteer F**	NT	NT	NT	NT	NT	NT	139

TNF-α production in the supernatant was determined after 4 hours of stimulation. The cytokine amounts shown are expressed as a percentage of the cytokine induction by whole cell *E. coli* preparations. NT = not tested.

## Discussion

The role of *S. wadsworthensis* in human gastrointestinal diseases has been documented in the past but its specific involvement in inflammatory bowel disease has never been firmly established [8], [9]. The fortuitous isolation of this unusual bacterial strain from mucosal biopsies of patients with IBD prompted a series of experiments that have been outlined in this paper and has culminated in detailed characterization of this putative pathogen. Despite this detailed microbial analysis no specific differences have been identified between cases and controls. However, this negative result does not preclude the importance of successful isolation of this fastidious organism from colonic biopsy samples by adhering to a strict microaerophilic culture environment and stringent growth conditions. Our study provides an important proof of concept that will enable future studies to satisfy Koch's postulates to tie in other luminal microaerophilic bacteria as causative factors in IBD.

This is the first report of the appearances of this bacterial species under scanning electron microscopy (SEM). As opposed to previous descriptions, the bacteria are pleomorphic, with strains existing in predominantly two morphological forms, long rods and coccobacilli. Filamentous and helical forms were also documented. The bacteria appeared in clusters on Gram stain and this was further corroborated with the appearance of a lattice type network on SEM. The functional role of this lattice formation is unclear, but given the high prevalence of this organism that we have demonstrated in the human colonic mucosa, further investigation of the interaction of *S. wadsworthensis* in the complex luminal microbiota may be of interest. This lattice structural network may paradoxically harbour either commensal bacteria or pathogenic bacterial strains in close juxtaposition to the epithelial surface with diametrically opposite consequences.

All *S. wadsworthensis* strains were negative for urease, catalase and oxidase, consistent with the initial description by Wexler et al [8]. The authors had described *S. wadsworthensis* as asaccharolytic, and certainly none of the sugar assimilation tests from the API kit were found to be positive [8]. As *S. wadsworthensis* is phenotypically closely related to the *Campylobacter* genus, the API Campy kit, specifically for the identification of *Campylobacter* species was trialed. This yielded more promising results. All of the *S. wadsworthensis* strains tested were positive for the EST, ArgA and AspA tests, meaning they possessed the enzymes esterase, L-arginine arylamidase and L-aspartate arylamidase respectively. Esterases are often required to breakdown dietary residue and are mostly derived from the intestinal microbiota, with *E. coli, Bifidobacterium* and *Lactobacillus* species all known to be sources [19]. Colonic esterases have been shown to release powerful antioxidants, present in cereal bran, from complex ester-linked structures which could otherwise not be absorbed [20]. It is possible that the esterases produced by *S. wadsworthensis* have a similar role.

This study utilized a newly developed PCR to detect the presence of *S. wadsworthensis* in a subset of adult patients with UC and adult control patients. The rates of detection was similar in both these groups (83.8% vs. 86.1%, p = 0.64), suggesting that this bacterial strain is probably a commensal. The only other study that has looked at the prevalence of this pathogen in patients with gastrointestinal disease was by Engberg et al. who used PCR and DNA sequencing of the 16S rRNA gene to characterize bacterial populations in fecal samples [21]. They detected *S. wadsworthensis* in only 7 out of 1483 (0.47%) patients with GI disorders, and only 1 out of 107 (0.93%) healthy individuals. This wide disparity in prevalence between this study and our cohort of patients and controls suggests that *S. wadsworthensis* is closely adherent to the mucosal lining and is more likely to be identified from biopsy samples as opposed to feces. Additionally, this newly designed PCR for this bacterial strain was validated using the type strain of *S. wadsworthensis* as well as several related bacterial strains and will form a useful tool in future studies.

Despite similar rates of detection of *S. wadsworthensis* from patients with IBD and controls, our clinical isolates gave us an excellent opportunity to further characterize this organism. Several bacterial species have been noted to have distinctly different phenotypic and genotypic profiles when isolated from patients with clinical disease as opposed to controls. This has been recently documented in *Campylobacter concisus* and mucosa associated *E. coli* wherein distinct genomospecies have been documented with varying pathogenic potential [22], [23]. Sequence analysis of strains identified during the study from cases and controls did not cluster in distinct groups confirming that this phenomenon does not extend to *S. wadsworthensis*.

Proteomic analysis of *S. wadsworthensis* strains utilizing MALDI-TOF MS is being reported for the first time in this paper. MALDI-TOF MS has recently emerged as an alternative to phenotypic and genotypic methods for the fast and reliable identification of microorganisms down to the species level [16], [24], [25], [26]. It can be used to classify closely related bacteria, which may be indistinguishable by conventional methods [24]. It was therefore employed to investigate the diversity of the *S. wadsworthensis* protein profiles. A characteristic pattern was clearly demonstrated on the analysis of *S. wadsworthensis* strains, with a dominant peak at approximately 9400Da. Although no major differences were detected between the strains, MALDI-TOF MS proved to be a highly sensitive and reproducible method for the characterization of the bacterial mass spectra. Very small mass of sample were required, which was ideal for analyzing the slow and modest growing *S. wadsworthensis*. This finding will provide a useful reference for future studies and an alternative to genotypic methods like 16S rRNA gene sequencing.

The *in-vitro* cytokine analysis after monocyte challenge failed to show any distinct differences between strains isolated from patients with IBD and controls. This last finding essentially closes the loop in the series of studies looking at the possible role of *S. wadsworthensis* in IBD. Our experiments have conclusively shown that the prevalence of this bacterium is similar in IBD patients and controls and that there is no phenotypic, genotypic, proteomic or pathogenic characteristic to distinguish bacteria isolated from these two groups of patients. It is quite likely that *S. wadsworthensis* is a commensal and a harmless bystander amidst the inflammatory cascade that is typical of inflammatory bowel disease.
